# Benign prostatic hyperplasia and subsequent risk of bladder cancer

**DOI:** 10.1038/sj.bjc.6603730

**Published:** 2007-05-01

**Authors:** D Kang, A P Chokkalingam, G Gridley, O Nyren, J E Johansson, H O Adami, D Silverman, A W Hsing

**Affiliations:** 1 Division of Cancer Epidemiology and Genetics, National Cancer Institute, Bethesda, Maryland, USA; 2 College of Medicine, Seoul National University, Seoul, Korea; 3 School of Public Health, University of California, Berkeley, California, USA; 4 Department of Medical Epidemiology and Biostatistics, Karolinska Institute, Stockholm, Sweden; 5 Department of Urology and Clinical Medicine and Center for Assessment of Medical Technology, Obrebro University Hospital, Obrebro, Sweden

**Keywords:** benign prostatic hyperplasia, prostate cancer, population-based, standardised incidence ratio

## Abstract

We evaluated the risk of bladder cancer in a cohort of 79 280 Swedish men hospitalised for benign prostatic hyperplasia (BPH), identified in the Swedish Inpatient Register between 1964 and 1983 and followed until 1989 via multiple record linkages with nationwide data on cancer registry, death and emigration. Standardised incidence ratios (SIRs), the ratios of the observed to the expected numbers of incident bladder cancers, were used to calculate the risk associated with BPH. The expected number was calculated by multiplying the number of person-years by the age-specific cancer incidence rates in Sweden for each 5-year age group and calendar year of observation. Analyses were stratified by BPH treatment, latency, calendar year and presence of genitourinary (GU) comorbid conditions. After excluding the first 3 years of follow-up after the index hospitalisation, we observed 506 incident bladder cancer cases during follow-up in the cohort. No overall increased risk of bladder cancer was apparent in our main analysis involving the entire BPH cohort. However, among BPH patients with transurethral resection of the prostate (TURP), there was an increased risk in all follow-up periods; SIRs of bladder cancer during years 4–6 of follow-up was 1.22 (95% confidence interval=1.02–1.46), 1.32 for 7–9 years of follow-up, and 1.47 for 10–26 years of follow-up. SIRs of bladder cancer among TURP-treated BPH patients were particularly elevated among those with comorbid conditions of the GU tract (e.g., stone, infection, etc.); 1.72, 1.74 and 2.01 for 4–6, 7–9, 10–26 years of follow-up, respectively, and also for those whose diagnoses occurred before 1975, when TURP was more likely to be performed by a urologist than a general practitioner: 1.87, 1.90 and 1.74, respectively. These findings suggest that BPH overall is not associated with bladder cancer risk. However, among men treated with TURP, particularly those with other comorbid GU tract conditions, risk of bladder cancer was elevated.

Benign prostatic hyperplasia (BPH) is a common condition resulting in about 200 000 transurethral resections of the prostate (TURP) annually in the United States (Graves and Kozak, 1998). Autopsy studies show anatomic or microscopic evidence of BPH in approximately 20% of men aged 40–50 years, increasing to about 80% prevalence in men 70–80 years of age; however, only 25–50% of men with microscopic BPH present with clinical manifestations (Ziada *et al*, 1999).

BPH progression may cause lower urinary tract damage. Clinical manifestation of obstruction from such damage includes increased urinary symptoms, urodynamic evidence of reduced flow rate, and increased post-void residual volume (Djavan *et al*, 2002). If left untreated, a number of sequelae can occur, including acute urinary retention, recurrent urinary tract infections, hydronephrosis and even renal failure (Jacobsen *et al*, 2001).

Urinary retention resulting from untreated BPH may be a risk factor for bladder cancer. A study of beagle dogs suggested that the frequency of urination is inversely associated with the level of potential carcinogens in the urothelium (Kadlubar *et al*, 1991). In humans, a prospective study showed that a high fluid intake may be associated with a decreased risk of bladder cancer in men (Michaud *et al*, 1999). Thus, we hypothesised that urinary retention or accumulation of residual urine caused or aggravated by BPH increases contact time between carcinogens and the urothelium, leading to increased bladder cancer risk. There have been very few epidemiological studies of the association between BPH and bladder cancer. Two case–control studies (Mommsen and Sell, 1983; Nakata *et al*, 1995) reported that BPH may be associated with increased bladder cancer risk (*n*=165 and 303 cases, respectively). To further clarify the role of BPH in bladder cancer prospectively, we examined the risk of this cancer in a large national cohort of 87 532 men hospitalised with BPH between 1964 and 1983 in Sweden. In stratified analyses, we separately evaluated risk among BPH patients with recorded complications of the outflow obstruction (stones, infections), which also are possible risk factors for bladder cancer (Chow *et al*, 1997).

## METHODS

### BPH cohorts

All patients whose records in the Swedish Inpatient Register (IPR) contained a discharge diagnosis code for BPH (International Classification of Diseases (ICD)-7 610.10 or ICD-8 600.09) between 1964 and 1983, and whose national registration numbers were complete and referable to a person alive and residing in Sweden at the time of entry (first hospital diagnosis of BPH in the IPR), were included in the cohort. A detailed description of this cohort, the record-linkage data, and the methods of follow-up used in the current study have been presented previously (Nyren *et al*, 1995; Chokkalingam *et al*, 2003). Of 87 532 patients with a discharge diagnosis of BPH, 79 280 were included in the study after excluding patients with any cancer diagnosed before the entry date (*n*=8252, about 20% of these were bladder cancer). Based on the type of BPH treatment received at entry into the cohort (index BPH diagnosis), the 79 280 patients were categorised into four groups: 21 995 (27.7%) had no surgery, 37 410 (47.2%) had TURP, 19 035 (24.0%) had transvesical adenomectomy (TA, open surgery for removal of the entire inner core of the prostate), and 840 (1.0%) had both TURP and TA. Because of small numbers, no subgroup analyses were conducted among the 840 patients who received both TURP and TA; they were included only in analyses of all BPH patients.

### Comorbid conditions of genitourinary tract

Up to six diagnoses can be entered in the IPR per hospital admission. To evaluate the joint effect of BPH and comorbid conditions of the genitourinary (GU) tract on bladder cancer development, we examined the following diagnoses that were identified at the initial BPH visit as GU comorbidities: ICD-7 codes 590.10–609.99 and ICD-8 codes 580.99–607.98. The most common comorbid GU conditions (>5%) were (in descending order): nonspecific urinary tract infections (*n*=2499, 14.4%), urethral stricture (*n*=1514, 8.7%), bladder stone (*n*=1258, 7.2%), and ureter stone (*n*=1068, *n*=6.2%).

### Follow-up: bladder cancer ascertainment

Incident bladder cancer cases diagnosed between 1964 and 1989 were identified by record linkage of the IPR data to the Swedish Cancer Registry based on national registration numbers.

### Statistical analysis

Standardised incidence rates (SIR) were calculated as the ratio of the observed to the expected numbers of events, computed by multiplying the number of person-years at risk by age-specific cancer incidence rates in Sweden for each 5-year age group and calendar year of observation. Person-years at risk were calculated from the admission date for the hospital visit during which BPH was first recorded to the date of first cancer (any, including bladder cancer), emigration, death or end of the observation period (December 31, 1989), whichever occurred first after BPH diagnosis. However, because presence of an undiagnosed cancer is likely to increase the probability of hospital admission for another diagnosis, such as BPH, and as the screening involved in the work-up of patients with BPH may also increase the detection of cancer during the first few years of follow-up, we reframed from interpreted person-time and cancer occurrences in the first 3 years following the index BPH hospitalisation. The 95% confidence interval (CI) of the SIR was calculated based on the assumption that the observed number follows a Poisson distribution (Bailar and Ederer, 1964). We computed *P*-values from a chi-statistic to test for trends in SIRs with increasing years of follow-up, and *P*-values from *χ*^2^ tests of homogeneity between various patient subgroups (Table 1 2 and 3). None of these *P*-values were statistically significant (*P*<0.05) (Breslow and Day, 1987).

## RESULTS

During the 26-year follow-up period (569 370 persons-years), 910 incident primary bladder cancer cases were identified (Table 1). Excluding the first 3 years of follow-up, 506 bladder cancer cases remained for further analysis. There was no significant increased risk of bladder cancer in BPH patients overall. However, nonsignificant, gradually increasing SIRs above 1 were observed for the follow-up periods 4–6, 7–9 and 10–26. In addition, BPH patients who underwent TURP had a significantly increased risk of bladder cancer in all follow-up periods; SIRs of bladder cancer for 4–6 years of follow-up was 1.22 (95% CI=1.02–1.46), 1.32 (95% CI=1.06–1.62) for 7–9 years of follow-up and 1.47 (95% CI=1.14–1.87) for 10–26 years of follow-up (*P* for trend=0.23). BPH patients without surgical treatment showed no excess risk, whereas those with transvesicular adenomectomy showed a nonsignificant decreased risk. The test for homogeneity among these cohorts was not statistically significant (*P*=0.66).

Table 2 shows risk in relation to BPH by presence of comorbid conditions of the GU tract (e.g., stones, infections, etc.) at the time of BPH diagnosis, as listed in the IPR. The SIR of bladder cancer after 10 or more years of follow-up was elevated in all BPH patients who had such GU comorbidities (SIR=1.42, 95% CI=0.99–1.96). In particular, risk was elevated in TURP-treated BPH patients with comorbid conditions of the GU tract, with significant SIRs of 1.72, 1.74 and 2.01, for 4–6, 7–9 and 10–26 years of follow-up, respectively (Table 2). However, the increasing trend in risk over follow-up time was not significant. Urethral stricture was the only specific comorbidity associated with a significantly increased risk, but this observation was based on only 18 bladder cancer cases. All other uncommon (prevalence <5%) GU comorbid conditions (e.g., pyelonephritis, pyelocystitis, renal cyst, cystitis, etc.) had nonsignificantly increased risk of bladder cancer (data not shown).

Table 3 shows risk in each treatment subgroup by calendar year of BPH diagnosis (<1975, 1975+), as TURP gradually replaced TA in Sweden during the 1970s and 1980s and was generally performed by urologists. We found that, although there was no significant association of BPH with bladder cancer overall by calendar year, the risk of bladder cancer was significantly elevated only in TURP-treated BPH cases diagnosed before 1975. The test for homogeneity of SIRs between the two time periods was not statistically significant (*P*=0.67).

## DISCUSSION

In this large cohort study in Sweden, we found no significant overall excess risk of bladder cancer among all BPH patients. After stratifying by type of BPH treatment (TURP, TA, no surgery), BPH patients treated with TURP, a less invasive surgical intervention, had a significantly increased risk of bladder cancer compared with the general Swedish population. However, there was no significant increased risk among BPH patients without surgery and a nonsignificantly decreased risk among BPH patients with TA, an open surgical procedure.

One of the reasons we set out to examine the risk of bladder cancer following diagnosis of BPH was the potential that through increased urinary retention and post-void residual urine, urinary symptoms often associated with BPH, the urothelium of the bladder may have increased contact and exposure to potential carcinogens. However, the absence of a significant association of bladder cancer risk with total BPH in this study does not support this hypothesis. Nevertheless, increased risks were observed for BPH patients treated with TURP and with GU comorbid conditions even after 10 years of follow-up, suggesting such symptoms may contribute, at least partly, to an increased risk of bladder cancer in these patients. Indeed, several GU conditions, such as urinary tract infections and stones, have been associated with an increased risk (Nakata *et al*, 1995).

Other possible reasons for the elevation in the TURP-treated BPH patients include surveillance or treatment selection bias. The probability of surveillance bias is likely to be small, as we focused on bladder cancer cases diagnosed at least 4 years after BPH diagnosis. Differences in risk across various treatment sub-cohorts suggest that factors related to selection of treatment may have influenced, in part, subsequent risk of bladder cancer among patients with BPH. General health may have been one such factor influencing selection of BPH treatment. In this study, the TA subcohort is generally healthiest, followed by the TURP group and then the non-surgical group, as evidenced by lower mortality rates from non-GU comorbidities, including cardiovascular disease, diabetes mellitus and respiratory disease (Blomqvist *et al*, 1997). In addition, the index hospital discharge diagnosis of BPH was the primary diagnosis for only 53% of non-surgical patients, compared with 89 and 88% of TA and TURP participants, respectively (Blomqvist *et al*, 1997), suggesting that those undergoing surgery were less likely to have other health concerns.

On the other hand, when the BPH diagnosis was only secondary, misclassification of BPH is likely. Compared with the primary diagnoses, the secondary BPH diagnoses were more often made by doctors who were not specialists in urology. The average severity of the clinical manifestation may also have been less compared with patients who had BPH as the primary diagnosis. If there is a true relationship between BPH and bladder cancer, possible misclassification of the condition in some patients and a generally less severe disease may have contributed to the absence of any observable association in non-operated BPH patients.

Among BPH patients undergoing TURP, we observed an increased risk among those first hospitalised for BPH before 1975, but not for those first hospitalised in the later time period, suggesting that earlier index years are associated with greater bladder cancer risk. This finding could result from long latency of bladder cancer development, which is supported by the finding that increased risk of bladder cancer was more pronounced in BPH cases with follow-up of greater than 10 years. Alternatively, it could result from changes in medical practice, such as differences in the recommendations for TURP over time (Blomqvist *et al*, 1997). In Sweden, the use of TURP grew over the study period (∼20% of BPH patients for pre-1975, ∼60% for post-1975).

Another possible explanation for the observed excess bladder cancer risk among TURP-treated BPH patients is the TURP procedure itself. Although TURP is regarded as the gold standard for the management of BPH, follow-up studies reveal failures and complications in 1–18% of patients (Mebust *et al*, 1989; Faul, 1993; Blomqvist *et al*, 1997; Borth *et al*, 2001). Compared with TA, TURP treatment may not completely excise the enlarged prostate, and it is possible that incomplete BPH treatment may increase the risk of BPH-related adverse effects, such as urinary retention, post-void residual urine, and other urinary symptoms, which may, in turn, increase the risk of bladder cancer. A complicated postoperative course, on the other hand, may give scope of increased surveillance with entailing detection bias. Further studies are needed to clarify the nature of the observed association.

This study has several strengths. First, unlike previous studies of BPH, which were of a case-control design, the prospective nature of this study rules out recall bias. Second, cancer case ascertainment was conducted by using the comprehensive national health record system in Sweden, which was virtually complete by the end of follow-up (Mattsson and Wallgren, 1984). Third, the population-based nature of the IPR ensures maximal representation of the study base. Fourth, we were able to utilise both surgical and diagnostic data collected in the IPR, including other comorbid conditions of the GU tract, to investigate reasons for the observed results. Finally, the large sample size and long duration of follow-up in this study provide good statistical power with which to observe an association between BPH and bladder cancer, if one exists.

There are a few limitations in our study. Potential confounders were incompletely adjusted for in the study, because we do not have information on some known bladder cancer risk factors (e.g., smoking, occupation). In addition, multiple comparisons and subgroup analysis could have led to chance findings. Also, it should be noted that we used first date of hospitalisation for BPH as a proxy for date of BPH diagnosis, although it is clear that the diagnosis and onset of BPH can take place before the first hospitalisation for BPH. Because of the nature of in-patient data, only BPH patients who needed treatment were included in this nation-wide database. We were not able to include less severe BPH patients, thereby limiting our generalisability. Furthermore, because we identified our subcohorts based on their BPH treatment noted on the first hospitalisation for BPH (entry into the cohort), a small amount of person time attributed to the non-surgical subcohorts should have been distributed to the surgical subcohorts. The reason for our analytical technique was to avoid the forward bias (Lubin and Gail, 1984). However, as only 252 of the 12 502 BPH patients during the 26-year follow-up belong to this group, the extent of misclassification of person-time is minimal.

In conclusion, this large cohort study indicated that BPH did not influence subsequent risk of bladder cancer overall. However, among men treated with TURP, a less invasive surgical intervention, risk of bladder cancer was elevated, particularly among men with other comorbid GU tract conditions.

## Figures and Tables

**Table 1:** Standardised incidence ratio (SIR)^a^ and 95% confidence intervals (CIs) for bladder cancer among patients diagnosed with benign prostatic hyperplasia in Sweden, 1964–1983

	**All BPH patients**			**No surgery**			**TURP**			**TA**		
**Follow-up years**	**Obs**	**Exp**	**SIR (95% CI)**	**Obs**	**Exp**	**SIR (95% CI)**	**Obs**	**Exp**	**SIR (95% CI)**	**Obs**	**Exp**	**SIR (95% CI)**
	(*n*=79 280)			(*n*=21 995)			(*n*=37 410)			(*n*=19 035)		
1	215	70.7	3.04 (2.65–3.47)	84	19.0	4.42 (3.53–5.47)	94	34.2	2.75 (2.22––3.37)	33	16.8	1.96 (1.35–2.75)
2	105	71.0	1.48 (1.21–1.79)	26	16.9	1.54 (1.01–2.26)	49	35.6	1.38 (1.02–1.82)	26	17.8	1.46 (0.95–2.14)
3	84	69.0	1.22 (0.97–1.51)	15	14.9	1.01 (0.56–1.66)	49	35.5	1.38 (1.02–1.83)	20	17.9	1.12 (0.68–1.73)
4	70	66.8	1.05 (0.82–1.32)	14	13.2	1.06 (0.58–1.78)	38	35.0	1.09 (0.77–1.49)	17	17.8	0.95 (0.56–1.53)
5–6	130	125.9	1.03 (0.86–1.23)	11	22.5	0.49 (0.24–0.87)	87	67.1	1.30 (1.04–1.60)	25	34.8	0.72 (0.47–1.06)
7–8	120	101.3	1.18 (0.98–1.42)	14	17.0	0.83 (0.45–1.39)	72	51.7	1.39 (1.09–1.75)	30	31.2	0.96 (0.65–1.37)
9–10	70	68.6	1.02 (0.80–1.29)	16	12.1	1.33 (0.76–2.16)	37	30.2	1.22 (0.86–1.69)	16	25.1	0.64 (0.36–1.04)
11–15	95	81.5	1.17 (0.94–1.42)	19	15.9	1.20 (0.72–1.87)	42	27.3	1.54 (1.11–2.08)	27	36.4	0.74 (0.49–1.08)
16–26	21	21.8	0.96 (0.60–1.47)	3	5.3	0.56 (0.11–1.64)	6	4.7	1.29 (0.47–2.80)	12	11.0	1.09 (0.56–1.90)
												
	(*n*=61 312)			(*n*=12 502)			(*n*=31 478)			(*n*=16,565)		
4–6	200	192.7	1.04 (0.90–1.19)	25	35.7	0.70 (0.45–1.03)	125	102.1	1.22 (1.02–1.46)	42	52.6	0.80 (0.58–1.08)
7–9	155	139.1	1.11 (0.95–1.30)	24	23.5	1.02 (0.65–1.52)	91	69.0	1.32 (1.06–1.62)	35	44.6	0.79 (0.55–1.09)
10–26	151	134.1	1.13 (0.95–1.32)	28	26.7	1.05 (0.70–1.52)	66	44.9	1.47 (1.14–1.87)	50	59.2	0.85 (0.63–1.11)

Abbreviations: BPH=benign prostatic hyperplasia; TURP=transurethral resection of the prostate; TA=transvesicular adenomectomy.

a Adjusted for age (in 5-year intervals) and calendar time period (in 5-year intervals).

**Table 2:** Standardised incidence ratio (SIR)^a^ and 95% confidence intervals (95% CI) for bladder cancer among patients diagnosed with benign prostatic hyperplasia in Sweden, 1964–1983 by comorbidity^b^ of genitourinary tract conditions

		**All BPH patients**				**BPH without surgery**				**BPH with TURP**				**BPH with TA**			
**GU Comorbidity**	**Follow-up**	**Obs**	**Exp**	**SIR**	**95% CI**	**Obs**	**Exp**	**SIR**	**95% CI**	**Obs**	**Exp**	**SIR**	**95% CI**	**Obs**	**Exp**	**SIR**	**95% CI**
No		(*n*=50 571)				(*n*=9803)				(*n*=26 072)				(*n*=14 211)			
	4–6	158	160.1	0.99	(0.84–1.15)	18	28.2	0.64	(0.38–1.01)	96	85.2	1.13	(0.91–1.38)	36	45.2	0.80	(0.56–1.10)
	7–9	123	115.1	1.07	(0.89–1.27)	17	18.5	0.92	(0.53–1.47)	70	57.0	1.23	(0.96–1.55)	31	38.4	0.81	(0.55–1.15)
	10–26	115	108.7	1.06	(0.87–1.27)	22	21.1	1.04	(0.65–1.58)	46	35.0	1.32	(0.96–1.76)	42	50.8	0.83	(0.60–1.12)
																	
Yes		(*n*=10 741)				(*n*=2699)				(*n*=5406)				(*n*=2354)			
	4–6	42	32.6	1.29	(0.93–1.74)	7	7.5	0.93	(0.37–1.91)	29	16.8	1.72	(1.15–2.47)	6	7.4	0.81	(0.30–1.77)
	7–9	32	24.0	1.33	(0.91–1.88)	7	5.0	1.41	(0.56–2.90)	21	12.1	1.74	(1.08–2.66)	4	6.2	0.64	(0.17–1.65)
	10–26	36	25.4	1.42	(0.99–1.96)	6	5.6	1.07	(0.39–2.32)	20	10.0	2.01	(1.23–3.10)	8	8.4	0.95	(0.41–1.88)
*P* c				*0.68*				*0.77*				*0.50*				*0.74*	

Abbreviations: BPH=benign prostatic hyperplasia; TURP=transurethral resection of the prostate; TA=transvesicular adenomectomy.

a Adjusted for age.

b Comorbid conditions include nonspecific urinary tract infections, urethral stricture, bladder stone, ureter stone.

c *P*-value for heterogeneity between SIR for no comorbidity *vs* with comorbidity within each subcohort.

**Table 3:** Standardised incidence ratio (SIR)^a^ and 95% confidence intervals (95% CI) for bladder cancer among patients diagnosed with benign prostatic hyperplasia in Sweden, 1964-1983 by year of BPH diagnosis

		**All BPH patients**				**BPH without surgery**				**BPH with TURP**				**BPH with TA**			
	**Follow-up**	**Obs**	**Exp**	**SIR**	**95% CI**	**Obs**	**Exp**	**SIR**	**95% CI**	**Obs**	**Exp**	**SIR**	**95% CI**	**Obs**	**Exp**	**SIR**	**95% CI**
<1975		(*n*=16 492)				(*n*=5249)				(*n*=3630)				(*n*=7278)			
	4–6	51	44.4	1.15	(0.86–1.51)	9	13.3	0.68	(0.31–1.28)	18	9.6	1.87	(1.11–2.95)	20	20.5	0.97	(0.59–1.50)
	7–9	43	38.7	1.11	(0.80–1.50)	11	10.0	1.10	(0.55–1.96)	17	9.0	1.90	(1.10–3.04)	13	18.8	0.69	(0.37–1.18)
	10–26	93	81.2	1.14	(0.92–1.40)	20	19.1	1.05	(0.64–1.62)	33	19.0	1.74	(1.19–2.44)	35	40.6	0.86	(0.60–1.20)
																	
1975–1983		(*n*=44 820)				(*n*=7253)				(*n*=27 848)				(*n*=9287)			
	4–6	149	148.3	1.00	(0.85–1.18)	16	22.4	0.71	(0.41–1.16)	107	92.4	1.16	(0.95–1.40)	22	32.0	0.69	(0.43–1.04)
	7–9	112	100.4	1.12	(0.92–1.34)	13	13.5	0.96	(0.51–1.65)	74	60.1	1.23	(0.97–1.55)	22	25.7	0.85	(0.54–1.29)
	10–26	58	52.9	1.10	(0.83–1.42)	8	7.6	1.05	(0.45–2.08)	33	25.9	1.27	(0.88–1.79)	15	18.5	0.81	(0.45–1.34)
*P* b				*0.70*				*0.27*				*0.49*			*0.94*		
*P* c						*0.67*											

Abbreviations: BPH=benign prostatic hyperplasia; TURP=transurethral resection of the prostate; TA=transvesicular adenomectomy.

a Adjusted for age.

b *P*-value for homogeneity test between calendar time periods.

c *P*-value for overall homogeneity test between two calendar time periods and three treatment methods (three subcohorts).
